# Local temperature-sensitive mechanisms are important mediators of limb tissue hyperemia in the heat-stressed human at rest and during small muscle mass exercise

**DOI:** 10.1152/ajpheart.00078.2015

**Published:** 2015-04-27

**Authors:** Scott T. Chiesa, Steven J. Trangmar, Kameljit K. Kalsi, Mark Rakobowchuk, Devendar S. Banker, Makrand D. Lotlikar, Leena Ali, José González-Alonso

**Affiliations:** ^1^Centre for Sports Medicine and Human Performance, Brunel University London, Uxbridge, UK; and; ^2^Department of Anaesthetics, Ealing Hospital NHS Trust, Southall, UK

**Keywords:** leg blood flow, heat stress, exercise

## Abstract

Limb tissue and systemic blood flow increases with heat stress, but the underlying mechanisms remain poorly understood. Here, we tested the hypothesis that heat stress-induced increases in limb tissue perfusion are primarily mediated by local temperature-sensitive mechanisms. Leg and systemic temperatures and hemodynamics were measured at rest and during incremental single-legged knee extensor exercise in 15 males exposed to 1 h of either systemic passive heat-stress with simultaneous cooling of a single leg (*n* = 8) or isolated leg heating or cooling (*n* = 7). Systemic heat stress increased core, skin and heated leg blood temperatures (T_b_), cardiac output, and heated leg blood flow (LBF; 0.6 ± 0.1 l/min; *P* < 0.05). In the cooled leg, however, LBF remained unchanged throughout (*P* > 0.05). Increased heated leg deep tissue blood flow was closely related to T_b_ (*R*^2^ = 0.50; *P* < 0.01), which is partly attributed to increases in tissue V̇O_2_ (*R*^2^ = 0.55; *P* < 0.01) accompanying elevations in total leg glucose uptake (*P* < 0.05). During isolated limb heating and cooling, LBFs were equivalent to those found during systemic heat stress (*P* > 0.05), despite unchanged systemic temperatures and hemodynamics. During incremental exercise, heated LBF was consistently maintained ∼0.6 l/min higher than that in the cooled leg (*P* < 0.01), with LBF and vascular conductance in both legs showing a strong correlation with their respective local T_b_ (*R*^2^ = 0.85 and 0.95, *P* < 0.05). We conclude that local temperature-sensitive mechanisms are important mediators in limb tissue perfusion regulation both at rest and during small-muscle mass exercise in hyperthermic humans.

upon exposure to acute heat stress, numerous cardiovascular adjustments occur in the human body to redistribute blood from the core to the peripheral tissues and increase peripheral blood flow, thereby aiding heat dissipation to the surrounding environment. At a systemic level, the increased demand for blood flow to the skin and outer extremities to dissipate heat is predominantly achieved through significant increases in cardiac output from ∼6 l/min to values as high as 12 l/min (33, 42, 48, 49, 53). These increases in cardiac output are primarily mediated through a significantly increased heart rate, with stroke volume remaining relatively stable (33, 49) due to an augmented ejection fraction (53) in the face of reduced central blood volume (11) and end-diastolic volume (53). At the peripheral level, increased tissue perfusion in the extremities is well documented and has been shown in the forearm (3, 8, 12, 13, 19, 24, 45), leg (22, 26, 42), and head (40). It remains unknown, however, whether the observed increases in cardiac output drive the increased perfusion evident in the extremities, or whether tissue blood flow responses to heat stress are determined by local temperature-sensitive mechanisms.

Numerous studies have provided compelling evidence that changes in peripheral tissue perfusion can be controlled by local regulatory mechanisms both at rest and during exercise. During exercise, studies using right atrial-pacing to manipulate the heart rate response to exercise in dogs (20, 21, 52) and more recently humans (1, 35) have shown that the tight coupling between oxygen demand and blood flow in skeletal muscle during exercise is chiefly regulated at a peripheral level through local mechanisms, with systemic adjustments such as the exercise-induced increase in heart rate occurring as a secondary response to regulate cardiac output. Taken together, these findings suggest that the combination of metabolic and temperature stimuli during the additive stresses of exercise and heat stress should result in an elevated leg blood flow to satisfy the demands for both metabolism and thermoregulation. Despite this, numerous studies have found no changes between normothermia and hyperthermia when performing single-leg knee extensions (14, 51), two-legged cycling (51), or uphill walking (37), whereas intense exercise in the heat with the added stress of dehydration causes the opposite effect with a decreased flow to the limb (18). In contrast, more recent work from our laboratory has shown an elevated leg blood flow when single-legged knee extensions were performed in the heat at a mild exercise intensity (42). However, whether these increases were due to a combination of local and systemic hemodynamic alterations and whether the response is maintained at higher exercise intensities remains to be determined.

The aims of this study, therefore, were twofold. First, by using two separate within-subjects contralateral limb models, we sought to identify the contribution of peripheral versus central thermosensitive mechanisms in the control of limb blood flow during heat stress by systematically altering leg tissue temperatures under conditions of both systemic heat stress and normothermia. Second, we aimed to assess the effect of limb temperature on leg blood flow during one-legged knee-extensor exercise up to levels approaching that of maximal power output. We hypothesized that *1*) limb blood flow would be primarily regulated at a peripheral level, and that this response would be closely coupled to increases in local tissue and/or blood temperatures, and *2*) increased local temperatures would result in elevated leg blood flow throughout incremental single-legged exercise to near maximal power output.

## METHODS

### Ethical Approval

Informed written consent was obtained from each participant before commencing the study. All procedures were approved by the Brunel University London Research Ethics Committee (RE04-11) and conformed to the *Declaration of Helsinki*.

### Participants

Fifteen healthy males (age, 23 ± 4 years; height, 177 ± 4 cm; weight, 73 ± 7 kg) were recruited to participate in two studies (*Study 1*, *n* = 8; *Study 2*, *n* = 7). Participants abstained from alcohol, caffeine, and strenuous exercise in the 24 h leading up to the day of testing.

### Experimental Protocols

**Fig. 1.**
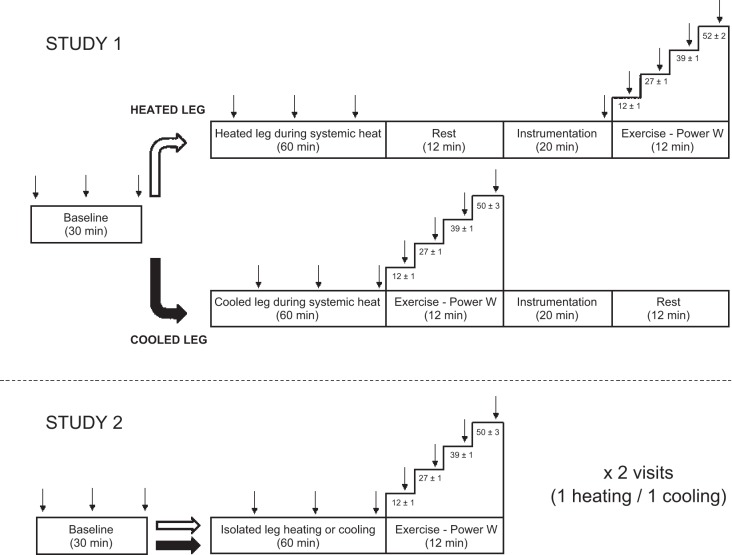
Sequence of the experimental protocols. In *Study 1*, participants were exposed to 1 h of passive whole-body heating through the use of a water-perfused suit, following 30 min of resting baseline measurements. The suit was designed to cover the entire body with the exception of the left leg, which was surrounded with frozen gel packs to cause isolated cooling of the limb. Immediately after the 1-h resting intervention, single-legged incremental knee-extensor exercise was carried out with either the cooled or heated limb, with each exercise protocol separated by at least 20 min. In *Study 2*, participants visited the laboratory on 2 occasions to have a single leg heated or cooled for 1 h, followed by an identical exercise bout that was carried out in *Study 1*. The order of heating and cooling was counterbalanced between visits. Arrows denote timing of measurements.

Two separate studies were conducted to investigate the role of local versus systemic effects of heat stress on lower limb tissue blood flow, its distribution, and potential underlying mechanisms at rest and during exercise. In *Study 1*, leg and systemic hemodynamic responses, temperatures, and blood variables were measured throughout 1 h of passive whole-body heat stress with single leg cooling before subsequent incremental single-leg knee extensor exercise was performed with both the cooled and heated legs (Fig. 1). Participants were passively heated through the use of a custom-built suit perfused with 50°C water and fitted to the entire upper body and right (heated) leg. Blood and tissue temperatures of the contralateral (cooled) leg were prevented from increasing via the application of frozen gel packs before being wrapped in an insulating blanket. Following the 1-h intervention, incremental single-leg knee extensor exercise (3-min stages) was performed with the cooled leg at 20%, 40%, 60%, and 80% peak power output (65 ± 3 W; determined during an earlier visit and identical for both left and right legs). Exercise was carried out on a custom-built modified Monark ergometer, with power outputs controlled within 6 W via an increased resistance on the flywheel following the application of metal weights. To account for slight variations in cadence, the individual work-rates for each stage were calculated by multiplying the average cadence by the weight causing the resistance on the flywheel. Following a 20-min rest period, the exercise protocol was then repeated with the heated leg at the same exercise intensities. In a follow-up study (*Study 2*), the isolated effects of limb heating and cooling were investigated by measuring leg and systemic hemodynamic and temperature responses in two separate visits involving 1 h of either isolated leg heating or cooling (Fig. 1). Following each intervention, single-leg knee extensor exercise was carried out as previously described for *Study 1*. Each laboratory visit for *Study 2* was separated by at least 1 wk and the order of heating and cooling was counterbalanced among participants.

### Instrumentation of Participants

In *Study 1*, participants reported to the laboratory at 8 a.m. following ingestion of their usual breakfast. Upon arrival, participants rested in the supine position to allow the ultrasound-guided placement of one double-lumen femoral intravenous catheter into each leg (Double Lumen Catheter, 18 gauge, 16 cm; Multi-Med M2716HE; Edwards Lifesciences) and one radial intra-arterial catheter into the right wrist under local anesthesia (1% lidocaine). Both femoral venous catheters were inserted ∼1 to 2 cm distal to the inguinal ligament and advanced in a retrograde direction (15 cm) to reside in the deep portion of the femoral vein. After successful placement, a fine-wire tissue implantable thermistor (PhysiTemp T-204A; Clifton, NJ) was advanced through the distal lumen of both femoral catheters to measure deep blood temperature within the femoral vein. Participants were then moved to the main experimental laboratory and placed in a supine position on the single-leg knee extensor ergometer (modified Monark ergometer; custom-built) with their legs rested on a table in front. Participants were fitted with a water-perfused suit for the manipulation of body temperature, designed to cover the entire upper body and right leg of the participant with the left leg remaining exposed to allow the application of ice packs for isolated limb cooling. The suit was connected to a thermostatically controlled water circulator (Julabo F-34; Seelbach, Germany) to allow the constant perfusion of 50°C water throughout the experimental protocol. A total of 7–9 ice packs (KoolPak, Warwickshire, UK) were secured to the cooled leg with Velcro strapping, with each pack being replaced after 30 min to prevent increases in leg temperature. A bag of crushed ice was also placed over the foot to provide almost complete coverage of the lower limb. Thus the ice packs and the bag of crushed ice provided almost continuous coverage of the entire limb, with only small spaces present between packs and around the knee. Participants were permitted to drink ad libitum throughout the protocol to prevent any confounding factors caused by dehydration. In *Study 2*, participants were positioned on the knee-extensor ergometer in a supine position in ambient conditions (20–22°C) and exposed to 1 h of isolated leg heating (single water-perfused leg cuff as mentioned before) or leg cooling (ice packs), with the contralateral leg acting as a control. In both studies, participants wore the water-perfused suit/leg cuff and ice packs throughout the entire duration of both rest and exercise protocols. Core, muscle (measured by fine-wire thermistor inserted 2 to 3 cm into the vastus lateralis), and skin temperatures; hemodynamic responses; and blood and plasma parameters were measured according to the procedures described below.

### Temperature Measurements

Core temperature (T_c_) was measured via the ingestion of a wireless telemetry pill (HQInc, Palmetto, FL; *Study 1*) or the self-insertion of a rectal thermocouple 15 cm beyond the anal sphincter (PhysiTemp, Clifton, NJ; *Study 2*). Mean body skin temperature of the whole body minus the cold leg (T̄_sk_) was calculated as a weighted mean using wireless temperature sensors (iButtons, Maxim, CA) attached to the arm, chest, thigh, and calf, with relative contributions calculated according to the formula T̄_sk_ = 0.3(T_chest_ + T_arm_) + 0.2(T_thigh_ + T_calf_). Blood temperature (T_b_) in *Study 1* was measured using the fine-wire thermocouples mentioned previously. Methodological limitations prevented the measurements of T_b_ in *Study 2*. However, measurements of muscle temperature (T_m_) obtained at a tissue depth of 2 to 3 cm allowed the assessment of the relationship between local tissue temperatures and blood flow. Leg skin temperature (T_sk leg_) was recorded via type-t thermocouples (PhysiTemp, Clifton, NJ) and calculated as the average of thigh and calf measurements. All temperature inputs were fed through a thermocouple meter (TC-2000; Sable Systems) for continuous measurement throughout the protocol.

### Hemodynamic Measurements

Leg blood flow (LBF) was measured both at rest and during single-legged exercise in the common femoral artery using a duplex Doppler ultrasound device (Vivid 7 Dimension; GE Medical, Horton, Norway) with a 10 MHz linear array transducer probe (GE Medical Systems UK). All measurements were taken at least 2 cm above the bifurcation into the superficial and profunda femoral arteries to minimize disruptions to measurements due to turbulent flow. Blood flow through the vessel was calculated as the product of the average arterial cross-sectional area obtained from three two-dimensional B-mode images and the mean velocity averaged over three 12-s Doppler scans (36 s total). Arterial diameter was consistently measured at peak systole (44), identified by an overlaid ECG trace. LBF was calculated in milliliters per minute using the equation: LBF = *V*_mean_ × π × (D/2)^2^ × 60, where *V*_mean_ is the time-averaged mean velocity of the blood expressed as centimeters per second, π is a mathematical constant, D is the diameter of the vessel in cm, and 60 is a constant used to convert the units to milliliters per minute. During the resting protocol, blood flow was also measured in both the superficial and profunda femoral arteries to characterize the distribution of flow to different portions of the leg.

Changes in skin blood flow during resting conditions were assessed noninvasively using laser Doppler flowmetry (Periflux 4001; Jarfalla, Sweden) via a 780-nm wavelength single-point laser Doppler probe (408, Periflux; Jarfalla, Sweden) fastened securely above the vastus lateralis muscle of each leg. In *Study 1*, mean arterial and femoral venous pressures (MAP and FVP, respectively) were measured directly from the radial and femoral venous catheters using pressure transducers at the level of the heart and leg (Pressure Monitoring Set; Edwards LifeSciences Germany) connected to two amplifiers (BPAmp; ADInstruments, Oxford, UK) and fed to a data acquisition system (PowerLab 16/30; AdInstruments, Oxford, UK). The participants' supine position allowed the blood pressure reference points to be essentially the same and thus perfusion pressure of the leg was calculated as MAP - FVP. MAP in *Study 2* was measured noninvasively using infrared photoplethysmography (Finometer, FMS, Netherlands).

In both studies, cardiac output (Q̇) was calculated as heart rate × stroke volume, with stroke volume estimated using the ModelFlow method (Beatscope, FMS, Netherlands) following corrections for participants' age, sex, mass, and height (57). Leg and systemic vascular conductance were calculated as common femoral artery/perfusion pressure and Q̇/MAP, respectively. Leg O_2_ delivery to each leg was calculated as LBF × arterial O_2_ content, while leg a-vO_2_ difference was calculated for both heated and cooled legs using the difference between arterial O_2_ content and heated and cooled femoral venous O_2_ contents, respectively. Due to the positioning of the sampling catheter beyond the saphenofemoral junction, whole-leg V̇O_2_ was calculated using the following modified two-component Fick equation: whole-leg V̇O_2_ = [(LBF − GSVBF) × (a-v_fdeep_ O_2_ difference)] + [GSVBF × (a-v_sk_ O_2_ difference)], where LBF is whole-leg blood flow, GSVBF is great saphenous vein blood flow (estimated using comparable data from a follow-up study; Chiesa et al. unpublished; see experimental considerations), a-v_fdeep_ O_2_ difference is the difference between radial arterial and deep femoral O_2_ content, and a-v_sk_ O_2_ difference is the difference between radial arterial and great saphenous vein oxygen content (the latter of which was calculated using [Hb] and PO_2_ measurements from the present study combined with estimates of superficial venous oxygen saturation from a previous study) (12).

### Blood Parameters

Arterial and femoral venous blood samples (1 ml each) were drawn into preheparinized syringes and analyzed immediately for blood gas variables, hemoglobin, electrolytes, lactate, and glucose (ABL 800 FLEX; Radiometer, Copenhagen, Denmark) with values corrected to blood temperatures measured simultaneously from the site of sampling in each vessel and the analyzer calibrated at regular intervals in accordance with manufacturer guidelines. Additional arterial and femoral venous blood samples from both legs were collected in 2-ml syringes and transferred to EDTA tubes, centrifuged, and separated. Plasma adrenaline and noradrenaline were subsequently determined using an enzyme-linked immunoassay kit (DEE6500 2-CAT; Demeditec Diagnostics GmbH, Kiel, Germany).

### Statistical Analysis

A one- and two-way repeated-measures ANOVA was used to test for differences within and between legs, with Holm-Bonferroni post hoc testing used to identify the time points at which changes occurred once a significant effect was found. Differences between studies were assessed using an independent samples two-way ANOVA with similar post hoc testing. Multiple regression for within-subject repeated measures was used for the analysis of the relationship between blood flow and blood gas variables and temperatures (4). All statistical analyses were carried out using SPSS (Version 20; IBM, Armonk, US) with results expressed as means ± SE. Significance is set at *P* < 0.05.

## RESULTS

### Hemodynamic Responses to Altered Leg Temperature During Systemic Heat Stress (Study 1)

#### Resting responses.

**Fig. 2.**
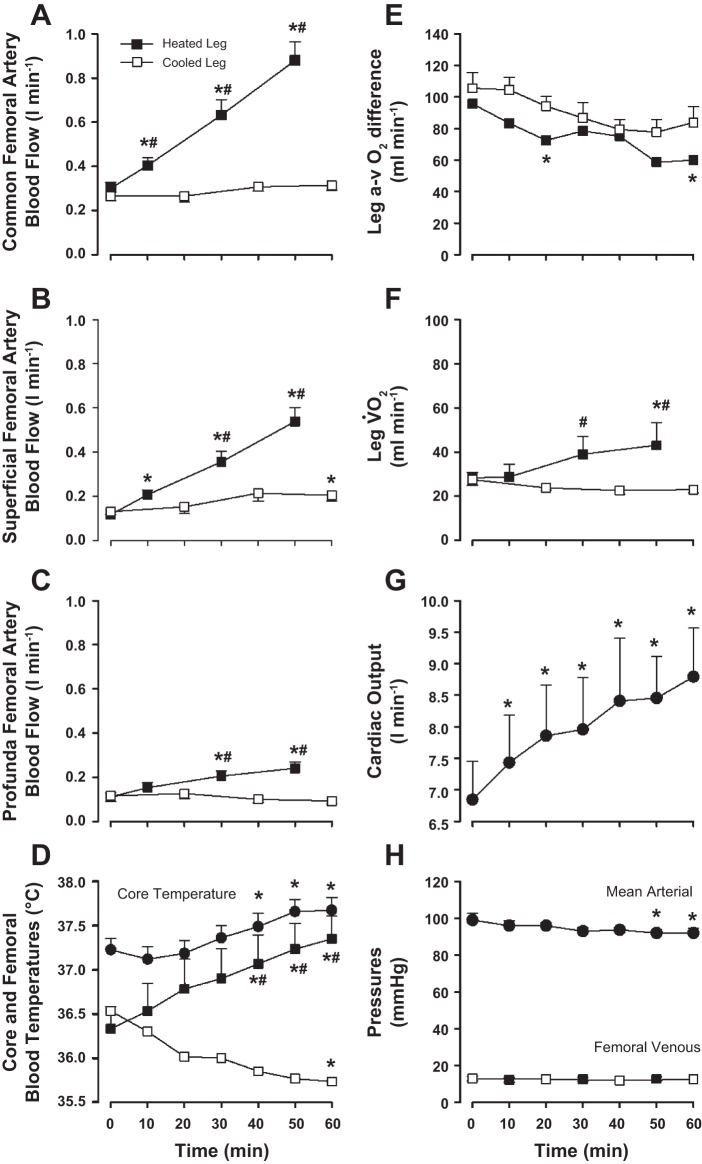
*A–H*: hemodynamic and temperature responses to systemic and leg heat stress combined with leg cooling (*Study 1*). Leg and systemic hemodynamic and temperature responses over 1 h of passive heat stress with simultaneous isolated cooling of a single leg are shown. Values are means ± SE for 8 participants except for cardiac output (n = 7) and core temperature (n = 6). *Significantly different from baseline; #significantly different from comparable measurement in the cooled leg. *P* < 0.05.

Full temperature responses are shown in Table 1. Systemic heating resulted in a 0.5 ± 0.1°C increase in T_c_ over the 1-h resting intervention, with an associated rapid and significant increase in T̄_sk_ from 32 to ∼38°C. Heated leg T̄_sk_ was significantly increased throughout to ∼38°C, whereas the corresponding T_b_ steadily increased from 36.3 ± 0.3 to 37.4 ± 0.3°C over 1 h (*P* < 0.05; Fig. 2*D*). In contrast, cooled leg T̄_sk_ decreased rapidly from 29 to ∼17°C; *P* < 0.05, and remained at this level for the duration of the test, whereas T_b_ also decreased to a level significantly below baseline (36.5 ± 0.2 to 35.7 ± 0.4°C; *P* < 0.05). Consequently, T̄_sk_ and T_b_ were significantly higher in the heated than the cooled leg from 40 to 60 min (∼12 and 1.7°C higher, respectively; *P* < 0.05).

**Table 1.** Temperature and hemodynamic responses to systemic and leg heating combined with leg cooling at rest and during exercise (Study 1)

	Systemic and Leg Heating with Single Leg Cooling		Exercise Cooled Leg		Exercise Heated Leg	
	Start	End	Start	End	Start	End
Systemic variables						
Core temperature, °C	37.2 ± 0.1	37.7 ± 0.1*	37.7 ± 0.1	37.8 ± 0.1	37.9 ± 0.1	38.0 ± 0.1
T̄_sk_, °C	32.0 ± 0.2	38.6 ± 0.2*	38.4 ± 0.1	38.3 ± 0.2	37.5 ± 0.1	37.6 ± 0.1
Heart rate, beats/min	69 ± 3	94 ± 4*	91 ± 4	150 ± 9*	93 ± 5	149 ± 9*
Stroke volume, ml	100 ± 7	90 ± 7	85 ± 7	86 ± 8	81 ± 7	88 ± 6
MAP, mmHg	99 ± 4	91 ± 3*	94 ± 4	141 ± 5*	92 ± 7	120 ± 1*
Leg variables						
T̄_sk_, °C						
Heated leg	28.9 ± 0.9	38.2 ± 0.9*#	—	—	36.3 ± 0.5	36.3 ± 0.6#
Cooled leg	29.1 ± 0.3	17.3 ± 0.2*	19.9 ± 1.4	23.8 ± 0.6*	—	—
Blood temperature, °C						
Heated leg	36.3 ± 0.3	37.4 ± 0.3*#	—	—	37.4 ± 0.2	37.7 ± 0.2*#
Cooled leg	36.5 ± 0.2	35.7 ± 0.4*	35.7 ± 0.4	36.3 ± 0.4*	—	—
SkBF, arbitrary units						
Heated leg	8 ± 2	69 ± 11*#	—	—	—	—
Cooled leg	9 ± 2	14 ± 6	—	—	—	—

Values are means ± SE for 8 participants for all variables except heart rate, stroke volume (*n* = 7), and core and blood temperatures and skin blood flow (*n* = 6).

* Significantly different from baseline;

# significantly different from cooled leg. *P* < 0.05.

In the heated leg, blood flow through the common femoral artery (i.e., LBF) showed a steady and significant increase throughout the duration of the 1-h intervention period (0.30 ± 0.03 to 0.88 ± 0.08 l/min; *P* < 0.01; Fig. 2*A*) due to a fourfold increase in local vascular conductance, which in turn was significantly correlated to increases in femoral venous blood temperature (*R*^2^ = 0.81: *P* < 0.01; Fig. 5). Similarly, blood flow through the superficial and profunda femoral arteries also displayed significant increases throughout, with the magnitude of increase being greater to the superficial artery (4.5-fold increase; 0.12 ± 0.01 to 0.54 ± 0.06 l/min; Fig. 2*B*) than the profunda artery (2-fold increase; 0.11 ± 0.02 to 0.24 ± 0.03 l/min; Fig. 2*C*). In the isolated cooled leg, no significant changes in flow were observed in any artery at the end of the 1-h intervention (*P* > 0.05). Skin blood flow in the heated leg increased from 8 ± 2 to 69 ± 11 AU (*P* < 0.01), whereas in the cooled leg values were not significantly different from baseline (final value 14 ± 6 AU; *P* > 0.05).

At the systemic level, Q̇ increased ∼1.9 l/min after 1 h of heating (*P* < 0.05; Fig. 2*G*), with the response owed solely to increases in heart rate (69 ± 3 to 94 ± 4 beats/min; *P* < 0.01) as stroke volume remained unchanged. A gradual decrease in MAP over the hour (99 ± 4 to 91 ± 3 mmHg; *P* < 0.01; Fig. 2*H*) accounted for a decreased perfusion pressure to both legs, since femoral venous pressures remained unchanged throughout (∼12 mmHg; Fig. 2*H*). Blood hemoglobin, arterial O_2_ content, and electrolytes remained unchanged in both legs throughout the resting protocol (Table 2), indicating that blood volume remained essentially unchanged throughout both rest and exercise. In contrast, an unchanged arterial O_2_ content coupled with a significantly increased LBF resulted in an increased O_2_ delivery to the heated compared with the cooled leg (end values, 185 ± 23 vs. 64 ± 5 ml/min). Leg V̇O_2_ was significantly elevated in the heated leg after 1 h (28 ± 3 to 43 ± 1 ml/min; *P* < 0.05; Fig. 2*F*), despite a decrease in the a-vO_2_ difference of blood draining from the deep veins of the thigh skeletal muscles (87 ± 12 to 60 ± 8 ml/l; *P* < 0.05; Fig. 2*E*). Cooled leg V̇O_2_ remained unchanged throughout (*P* > 0.05). The significant differences in flow between the heated and the cooled legs were accompanied by declines in glucose and lactate a-v gradients from baseline to the end of the 1-h thermal protocols (i.e., 0.8-0.7 to 0.5-0.3 mmol/l and -0.2 ± 0.3 to −0.1 ± 0.1 mmol/l for glucose and lactate a-v gradients, respectively; *P* < 0.05; Table 2). However, total glucose uptake over the 1-h thermal protocol was significantly higher in the heated than the cooled leg (13.9 ± 3.7 vs. 9.2 ± 2.9 mmol; *P* < 0.05), whereas total lactate release did not reach detectable levels. Arterial and venous plasma noradrenaline concentrations were also stable over the 1-h thermal intervention in the heated and cooled legs (mean range values of 3 to 4 nmol/l). However, arterial adrenaline decreased from 1.7 ± 0.8 to 0.8 ± 0.2 nmol/l (*P* < 0.05) over the 1 h, whereas femoral venous values in the heated and cooled leg remained stable (Table 2). Multiple regression analyses displayed strong linear relationships between femoral venous blood temperature and both profunda femoral artery blood flow and tissue V̇O_2_ over the full range of temperatures in the cooled and heated legs (*R*^2^ = 0.50 and 0.55 for T_b_ vs. profunda femoral arteries and V̇O_2_, respectively; *P* < 0.01 for both).

**Table 2.** Blood variable responses to systemic and leg heating combined with leg cooling at rest (Study 1)

	Time, min						
	0	10	20	30	40	50	60
Hb, g/l							
a	147 ± 3	147 ± 3	145 ± 3	148 ± 3	147 ± 4	149 ± 4	149 ± 5
vh	147 ± 3	143 ± 4	145 ± 3	147 ± 3	149 ± 4	150 ± 4	150 ± 4
vc	146 ± 3	145 ± 3	147 ± 4	149 ± 4	149 ± 4	149 ± 4	151 ± 3
O_2_ saturation, %							
a	98 ± 0.1	98 ± 0.1	98 ± 0.3	98 ± 0.1	98 ± 0.1	98 ± 0.2	98 ± 0.2
vh	53 ± 6	62 ± 5*	66 ± 4*	64 ± 4	67 ± 5*	65 ± 5	70 ± 4*
vc	50 ± 6	56 ± 6	60 ± 5	62 ± 5	65 ± 5	62 ± 3	60 ± 5
Po_2_, mmHg							
a	99 ± 3	101 ± 3	109 ± 6	103 ± 2	112 ± 6	100 ± 4	105 ± 5
vh	30 ± 3	35 ± 3	37 ± 3	36 ± 3	39 ± 4	38 ± 3	41 ± 4
vc	28 ± 3	31 ± 3	32 ± 3	32 ± 2	36 ± 4	31 ± 2	33 ± 3
CtO_2_, ml/l							
a	200 ± 4	198 ± 4	197 ± 3	200 ± 4	199 ± 4	201 ± 5	201 ± 5
vh	106 ± 11	121 ± 12*	132 ± 8*	130 ± 9	136 ± 12	134 ± 13	146 ± 11*
vc	100 ± 11	111 ± 12	122 ± 13	127 ± 11	134 ± 12	128 ± 10	127 ± 13
Pco_2_, mmHg							
a	39 ± 1	37 ± 3	40 ± 1	41 ± 1	40 ± 1	40 ± 1	40 ± 1
vh	49 ± 2	48 ± 1	48 ± 1	48 ± 1	47 ± 1	48 ± 1	47 ± 1
vc	50 ± 2	49 ± 2	48 ± 2	47 ± 2	46 ± 2	46 ± 2	47 ± 2
pH							
a	7.43 ± 0.01	7.42 ± 0.01	7.43 ± 0.01	7.43 ± 0.01	7.43 ± 0.01	7.43 ± 0.01	7.42 ± 0.01
vh	7.39 ± 0.01	7.39 ± 0.01	7.39 ± 0.01	7.39 ± 0.01	7.40 ± 0.01	7.39 ± 0.01	7.39 ± 0.01
vc	7.39 ± 0.01	7.39 ± 0.01	7.39 ± 0.01	7.40 ± 0.01	7.40 ± 0.01	7.40 ± 0.01	7.40 ± 0.01
Glucose, mmol/l							
a	6.0 ± 0.2	5.8 ± 0.2	5.8 ± 0.3	6.0 ± 0.2	6.1 ± 0.3	6.3 ± 0.3	6.3 ± 0.4
vh	5.3 ± 0.4	5.4 ± 0.3	5.6 ± 0.2	5.7 ± 0.2	5.8 ± 0.2	5.9 ± 0.3	6.1 ± 0.3
vc	5.1 ± 0.4	5.3 ± 0.3	5.5 ± 0.3	5.5 ± 0.2	5.7 ± 0.2	5.7 ± 0.2	5.9 ± 0.2
Lactate, mmol/l							
a	1.2 ± 0.3	1.2 ± 0.3	1.1 ± 0.2	1.1 ± 0.2	1.1 ± 0.2	1.3 ± 0.2	1.5 ± 0.3
vh	1.1 ± 0.1	1.1 ± 0.1	1.0 ± 0.1	1.1 ± 0.1	1.1 ± 0.1	1.2 ± 0.1	1.4 ± 0.2
vc	1.1 ± 0.1	1.0 ± 0.1	1.1 ± 0.1	1.2 ± 0.1	1.1 ± 0.1	1.1 ± 0.1	1.4 ± 0.2
Noradrenaline, nmol/l							
a	2.4 ± 0.9	—	—	3.4 ± 1.3	—	—	2.6 ± 0.6
vh	3.8 ± 0.5	—	—	3.2 ± 0.6	—	—	3.0 ± 0.7
vc	3.2 ± 0.7	—	—	4.2 ± 1.8	—	—	3.6 ± 0.7
Adrenaline, nmol/l							
a	1.7 ± 0.4	—	—	1.4 ± 0.9	—	—	0.8 ± 0.2*
vh	0.5 ± 0.2	—	—	0.7 ± 0.3	—	—	0.8 ± 0.4
vc	0.4 ± 0.1	—	—	0.9 ± 0.6	—	—	0.5 ± 0.1

Values are means ± SE for 8 participants (venous samples) and 7 participants (arterial samples). Catecholamines were measured at time points 0, 30, and 60 only (*n* = 5). a, arterial; vh, femoral venous heated leg; vc, femoral venous cooled leg. Po_2_, Pco_2_, and pH were corrected for changes in blood temperature.

* Significantly different from baseline; *P* < 0.05.

#### Exercise responses.

**Fig. 3.**
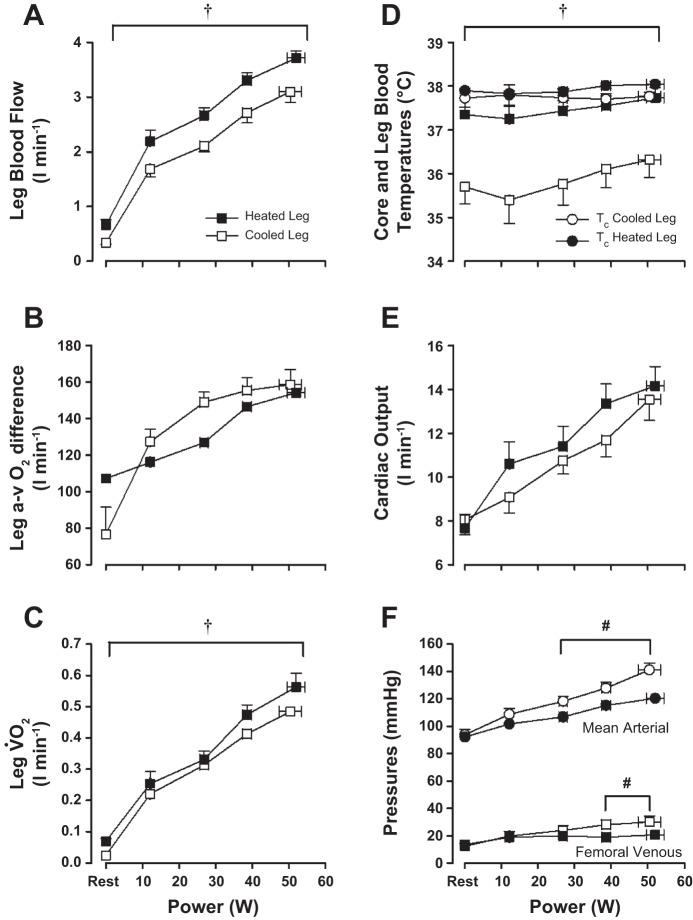
*A–F*: hemodynamic and temperature responses to exercise in the heated and cooled leg with systemic heat stress (*Study 1*). Leg and systemic hemodynamic and temperature responses during incremental single-legged knee extensor exercise at 20%, 40%, 60%, and 80% peak power output are shown. Participants were previously exposed to 1 h of full-body heat stress with simultaneous isolated single leg cooling before carrying out an incremental exercise test in both the heated and cooled limb. Values are means ± SE for 7 participants except for cardiac output (n = 6) and core temperature (T_c_; n = 5). †Mean effect for temperature (heated vs. cooled leg); #significantly different from cooled leg. *P* < 0.05.

No significant differences in power output were observed at any stage of incremental exercise between the heated and cooled legs (80% W_max_, 52 ± 2 vs. 50 ± 3 W; *P* = 0.48), whereas T_c_ and T̄_sk_ were also comparable throughout (∼38°C for both; *P* > 0.05). As expected, however, leg T̄_sk_ and T_b_ were significantly higher throughout the exercise bout with the heated than the cold leg (mean, ∼36 vs. 24°C for leg T̄_sk_ and 37.7 vs. 36.3°C for T_b_, respectively; *P* < 0.01; Table 1 and Fig. 3*D*). Blood flow through the common femoral artery of the heated leg (i.e., LBF) was consistently ∼0.6 l/min higher than that of the cooled leg at each stage of incremental exercise (final stage values, 3.7 ± 0.1 vs. 3.1 ± 0.2 l/min; *P* < 0.01; Fig. 3*A*), coinciding with an elevated leg vascular conductance of ∼6 ml·min^−1^·mmHg^−1^ during heated limb exercise in comparison with cooled. The differences in blood flow and vascular conductance between legs were once again closely associated with differences in femoral venous blood temperature draining each of the legs (*R*^2^ = 0.85 and 0.95 for heated and cooled legs, respectively; *P* < 0.01; Fig. 5). Blood hemoglobin, osmolality, and electrolytes showed no difference over both incremental exercise bouts (Table 3). No significant difference was observed in a-vO_2_ difference between the two legs, although results suggested that this tended to be lower over the duration of the heated leg exercise protocol (*P* = 0.059; Fig. 3*B*). The higher blood flow at each incremental stage, coupled with an unchanged arterial O_2_ content, resulted in increased O_2_ delivery in the heated compared with the cooled limb throughout the duration of exercise (*P* < 0.05). V̇O_2_ was significantly higher over the entire duration of the incremental exercise bout with the heated leg (Fig. 3*C*), coinciding with a decreased total lactate release (19.4 vs. 36.5 mmol; *P* < 0.05) and attenuated drop in venous pH (decrease of 0.11 vs. 0.19; *P* < 0.05; Table 3).

**Table 3.** Blood variable responses to exercise in the heated and cooled leg during systemic and leg heat stress combined with leg cooling (Study 1)

	Exercise With Cooled Leg, W					Exercise With Heated Leg, W				
	Rest	12 ± 1	27 ± 1	39 ± 1	50 ± 3	Rest	12 ± 1	27 ± 1	39 ± 1	52 ± 2
Hb, g/l										
a	156 ± 8	152 ± 4	151 ± 4	154 ± 4	152 ± 6	152 ± 4	153 ± 4	150 ± 6	152 ± 4	157 ± 4
vh	152 ± 4	152 ± 4	153 ± 4	152 ± 5	161 ± 3	150 ± 4	152 ± 5	149 ± 9	152 ± 6	152 ± 4
vc	152 ± 4	150 ± 4	153 ± 3	152 ± 4	161 ± 4*	153 ± 4	150 ± 5*	153 ± 10	155 ± 4	156 ± 3
O_2_ saturation, %										
a	98 ± 0.2	98 ± 0.3	98 ± 0.2	98 ± 0.2	98 ± 0.2	98 ± 0.4	98 ± 0.2	98 ± 0.4	98 ± 0.2	98 ± 0.2
vh	71 ± 4	61 ± 3	52 ± 4*	52 ± 4*	53 ± 5*	48 ± 6	43 ± 4	36 ± 4	28 ± 3*	27 ± 4*
vc	59 ± 7	37 ± 4*	26 ± 2*	25 ± 3*	21 ± 3*	66 ± 6	59 ± 5	56 ± 3*	52 ± 4*	48 ± 4*
Po_2_, mmHg										
a	105 ± 6	103 ± 4	101 ± 2	106 ± 4	109 ± 3	111 ± 6	100 ± 3	110 ± 10	102 ± 2	105 ± 2
vh	34 ± 1	34 ± 1	31 ± 1	32 ± 2	32 ± 2	30 ± 2	28 ± 2	26 ± 1	23 ± 1	23 ± 2
vc	32 ± 4	23 ± 1*	20 ± 1*	20 ± 1*	20 ± 1*	38 ± 4	32 ± 2	31 ± 2	30 ± 2	29 ± 2
CtO_2_, ml/l										
a	200 ± 4	205 ± 4	204 ± 5	208 ± 4	207 ± 7	206 ± 5	206 ± 5	202 ± 7	205 ± 4	212 ± 5
vh	138 ± 1	127 ± 1	110 ± 1*	108 ± 1*	116 ± 1*	99 ± 1	90 ± 1	75 ± 1*	59 ± 1*	58 ± 1*
vc	127 ± 2	78 ± 1*	55 ± 0.3*	53 ± 0.4*	48 ± 1*	135 ± 2	111 ± 2*	112 ± 1*	110 ± 1*	100 ± 1*
Pco_2_, mmHg										
a	41 ± 1	40 ± 1	42 ± 1	39 ± 2	38 ± 2	36 ± 3	39 ± 2	36 ± 2	39 ± 2	38 ± 2
vh	47 ± 1	49 ± 1	52 ± 1*	53 ± 1*	53 ± 2*	50 ± 2	55 ± 2*	59 ± 2*	64 ± 2*	71 ± 4*#
vc	47 ± 3	56 ± 3*	65 ± 3*	73 ± 4*	80 ± 3*	43 ± 2	44 ± 2	46 ± 2*	48 ± 1*	50 ± 1*
pH										
a	7.42 ± 0.01	7.41 ± 0.01	7.39 ± 0.01	7.39 ± 0.01	7.40 ± 0.01	7.41 ± 0.03	7.40 ± 0.01	7.41 ± 0.02	7.39 ± 0.01	7.39 ± 0.01
vh	7.39 ± 0.01	7.37 ± 0.01	7.36 ± 0.01*	7.35 ± 0.01*	7.34 ± 0.01*	7.36 ± 0.02	7.34 ± 0.01	7.31 ± 0.01*	7.29 ± 0.01*	7.25 ± 0.02*#
vc	7.40 ± 0.01	7.33 ± 0.01*	7.29 ± 0.01*	7.25 ± 0.02*	7.21 ± 0.02*	7.36 ± 0.02	7.37 ± 0.02	7.36 ± 0.01	7.36 ± 0.01	7.35 ± 0.01
Glucose, mmol/l										
a	6.5 ± 0.4	6.6 ± 0.3	6.6 ± 0.2	6.4 ± 0.2	6.3 ± 0.2	6.6 ± 0.2	6.7 ± 0.3	6.8 ± 0.3	6.8 ± 0.4	6.8 ± 0.4
vh	6.1 ± 0.3	6.1 ± 0.3	6.1 ± 0.2	6.1 ± 0.2	6.0 ± 0.3	6.1 ± 0.3	6.4 ± 0.5	6.3 ± 0.5	6.2 ± 0.6	6.1 ± 0.6
vc	5.9 ± 0.2	6.2 ± 0.3	6.3 ± 0.2	6.1 ± 0.2	6.3 ± 0.3	5.9 ± 0.4	6.2 ± 0.3	6.4 ± 0.4	6.6 ± 0.4	6.6 ± 0.4
Lactate, mmol/l										
a	1.5 ± 0.3	1.7 ± 0.3	2.2 ± 0.3*	3.0 ± 0.5*	4.1 ± 0.7*	3.6 ± 0.9	3.4 ± 0.7	3.4 ± 0.7	4.0 ± 0.4	4.7 ± 0.5*
vh	1.4 ± 0.2	1.4 ± 0.2	1.7 ± 0.2	2.2 ± 0.3*	3.3 ± 0.5*	2.9 ± 0.5#	3.4 ± 0.5#	3.5 ± 0.5*	4.0 ± 0.6*	5.3 ± 0.9*
vc	1.4 ± 0.2	2.3 ± 0.3*	3.0 ± 0.4*	4.3 ± 0.6*	6.0 ± 0.8*	4.4 ± 0.9	3.5 ± 0.7	3.4 ± 0.5	3.3 ± 0.5	3.8 ± 0.5

Values are means ± SE for 7 participants (venous samples) and 6 participants (arterial samples). Po_2_, Pco_2_, and pH were corrected for changes in blood temperature.

* Significantly different from baseline;

# significantly different from cooled femoral venous blood during cooled leg exercise. *P* < 0.05.

At the systemic level, heart rate (∼90 to 150 beats/min), stroke volume (∼85 ml throughout), and Q̇ (∼8 to 13 l/min) were similar during incremental exercise with the heated and the cooled leg (*P* > 0.05; Table 1 and Fig. 3*E*). MAP and FVP both significantly increased over the duration of the incremental exercise tests, with smaller increases in both being observed during exercise with the heated compared with the cooled leg (final values for MAP and FVP, 124 ± 4 vs. 141 ± 5 mmHg and 21 ± 2 vs. 30 ± 4 mmHg, respectively; *P* < 0.05; Fig. 3*F*).

### Hemodynamic Responses to Isolated Changes in Leg Temperature (Study 2)

#### Resting responses.

T_c_ and T̄_sk_ were maintained at 37° and 32°C throughout both isolated heating and cooling protocols, whereas heated and cooled leg T_sk_ were comparable with that obtained during *Study 1* (final values, 38.4 ± 0.9 and 19.5 ± 1.6°C, respectively). LBF responses to isolated heating were similar to that observed in the heated leg during systemic heat stress, with increases observed in the common (0.25 ± 0.02 to 0.76 ± 0.08 l/min), superficial (0.13 ± 0.01 to 0.46 ± 0.07 l/min), and profunda femoral arteries (0.08 ± 0.01 to 0.22 ± 0.05 l/min) (*P* < 0.01 for all). As in *Study 1*, these changes in LBF were once again associated with increasing local tissue temperatures (T_m_; R^2^ = 0.55; *P* < 0.01). Isolated cooling of the limb led to a small but significant decrease in flow to the common, superficial, and profunda femoral arteries (0.19 ± 0.01 to 0.16 ± 0.01 l/min, 0.09 ± 0.02 to 0.08 ± 0.01 l/min, and 0.07 ± 0.02 to 0.04 ± 0.01 l/min; *P* < 0.01). The difference in flow between the heated and cooled legs at the end of the 1-h intervention was similar to that observed during *Study 1* (∼0.6 l/min; *P* < 0.01). The increases in LBF with isolated heating were paralleled by increases in leg vascular conductance, which in turn was significantly correlated with T_m_ (*R*^2^ = 0.55; *P* < 0.01), but not T_c_ or T_sk_ (*R*^2^ = 0.07 and 0.37, respectively; *P* > 0.05).

#### Exercise responses.

**Fig. 4.**
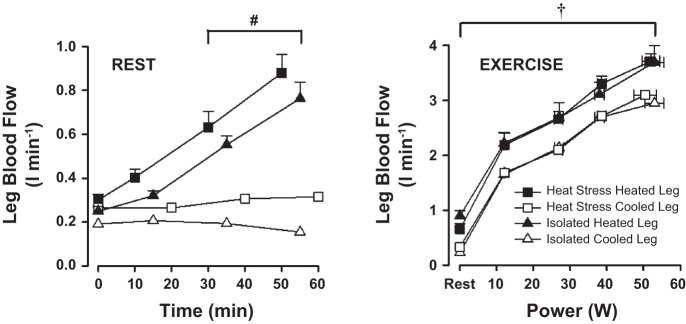
Leg blood flow responses to leg heating and cooling with and without systemic heat stress (*Study 1* and *Study 2*). Values are means ± SE for 8 participants during whole-body hyperthermia (exercise data, n = 7) and 7 participants during normothermia. †Mean effect for temperature (both heated vs. both cooled legs); #significantly different from both cooled legs. *P* < 0.05.

There were no differences in power output, T_c_, or T̄_sk_ during each incremental exercise test with either isolated leg heating or cooling (*P* > 0.05 for all). However, leg T̄_sk_ was - as expected - significantly elevated throughout incremental exercise in the heated compared with the cooled leg (∼37 and 15°C, respectively; *P* < 0.01). LBF of the heated leg was consistently ∼0.6 l/min higher than that of its cooled counterpart at each stage of incremental exercise and was not different between *Studies 1 and 2* (final stage values, ∼3.7 vs. 3.1 l/min; *P* < 0.01; Fig. 4*B*). Increases during incremental exercise with the heated and cooled legs were associated with progressive increases in T_m_.

### Hemodynamic Responses to Altered Leg Temperature With and Without Systemic Heat Stress: A Comparison of Both Studies

At rest, the effect of heating or cooling the leg with and without systemic heat stress resulted in similar LBF responses over the 1-h intervention, despite differences in systemic temperatures and hemodynamic responses. LBF in the heated leg at the end of *Study 1* was within ∼0.1 l/min of that recorded in *Study 2* (0.94 ± 0.1 vs. 0.80 ± 0.11 l/min; *P* > 0.05), with similar responses being observed in the cooled leg also (0.19 ± 0.02 vs. 0.33 ± 0.03 l/min; *P* > 0.05; Fig. 4*A*). These similar flows occurred despite significant differences in both T_c_ (37.7 vs. 37.1°C; *P* < 0.01) and Q̇ (8.8 ± 0.6 vs. 6.4 ± 0.5 l/min; *P* < 0.05) between the two studies.

During incremental exercise, LBF was determined by a combination of exercise intensity and local blood and/or tissue temperatures; with heated and cooled leg blood flows in the systemic heat stress incremental exercise tests showing no difference to their isolated heated and cooled leg conditions (Fig. 4*B*), despite differences in T_c_ of ∼1°C (final heated and cooled leg blood flows, ∼3.7 vs. 3.1 l/min during both heat stress and control conditions).

## DISCUSSION

This investigation sought to elucidate the contribution of local versus systemic thermosensitive mechanisms on global leg perfusion and its distribution in a variety of thermal and exercise conditions in healthy humans. A major finding was that increases in resting blood flow in the leg's largest conduit arteries in response to both isolated limb and systemic heat stress were strongly correlated to increases in local tissue and/or blood temperatures, but were unrelated to the distinct core temperature and systemic hemodynamic responses. Consistent with this notion, we also found that blood flow in the cooled leg's common, superficial, and profunda femoral arteries in *Study 1* remained essentially unchanged, despite physiologically significant increases in systemic temperature and blood flow with heat stress. The presence of an increased profunda femoral artery blood flow (main conduit vessel supplying blood to deep thigh tissues) is indicative of a contribution of thigh skeletal muscle hyperemia to the overall increase in limb tissue perfusion with heat stress, a response that may be partly attributable to increases in metabolically mediated vasodilatation. These increases in blood flow to leg tissues at rest are maintained during incremental single-legged knee-extensor exercise to near maximal power output. This suggests an additive effect of local thermoregulatory and metabolic stimuli on the regulation of leg perfusion during exercise activities engaging a small muscle-mass.

### Local and Systemic Influences on Leg Tissue Perfusion During Heat Stress

**Fig. 5.**
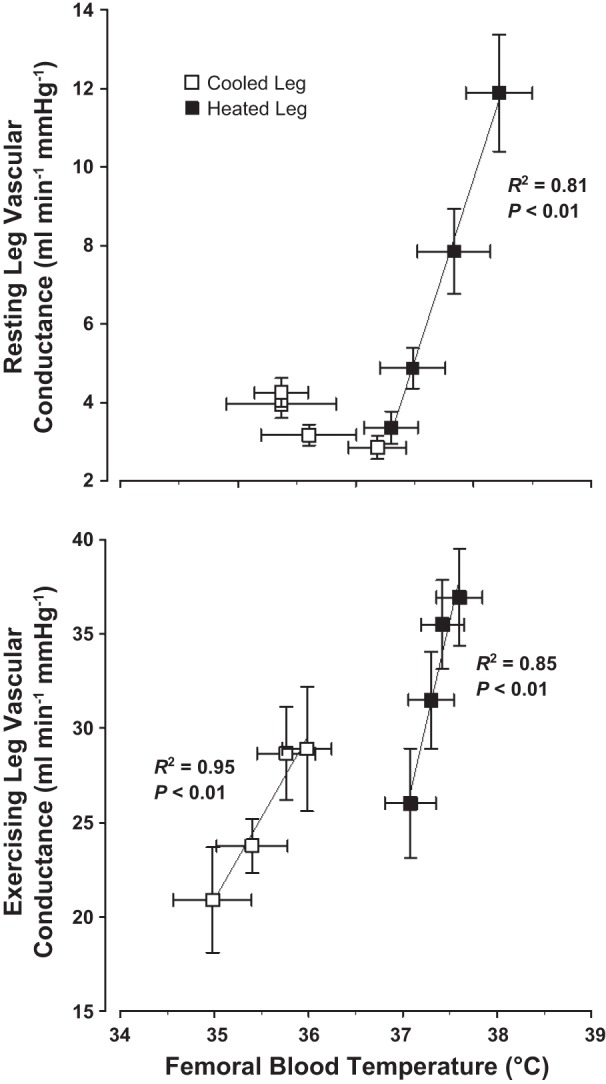
Relationship between leg vascular conductance and femoral venous blood temperature in cooled and heated legs during both rest (*top*) and exercise (*bottom*). Values are means ± SE for 8 participants in *Study 1*. *P* < 0.05 for all indicated relationships.

We systematically altered leg tissue temperatures under conditions of systemic and isolated limb thermal stress, with a major finding being the tight coupling between elevations in resting blood flow in the major conduit arteries of the leg and the increases in local tissue and/or blood temperatures, mechanistically dissociated from core temperature, systemic hemodynamic stimuli, and a myriad of arterial blood parameters. Several observations underpinned this novel finding. First, and in agreement with a recent study from our laboratory (42), we found significant increases in blood flow in the passively heated leg (0.6 l/min) during systemic heat stress, accompanied by increases in T_c_ (0.5°C), heart rate (30 beats/min), Q̇ (2 l/min), and decreased MAP and leg perfusion pressure (∼7%). The increased leg hyperemia in the face of a decreased perfusion pressure gradient was strongly associated with a corresponding elevation in leg vascular conductance, a response that was closely related to the rise in femoral venous blood temperature (*R*^2^ = 0.81; *P* < 0.01; Fig. 5), with weaker relationships observed with T_c_ (*R*^2^ = 0.67; *P* < 0.05) and T̄_sk_ (clamped at ∼38°C throughout the experiment; *R*^2^ = 0.45; *P* < 0.05). Second, an involvement of local temperature in the regulation of LBF was revealed by the prevention of the hyperemic response during the simultaneous cooling of the contralateral limb, despite the same increases in central hemodynamics and core temperature and the same concentration of neuro-humoral factors in the arterial vessels supplying both legs. Last, data during isolated heating of a single leg showed comparable LBF responses with that seen during systemic heat stress with a significant correlation observed between leg vascular conductance and T_m_ (*R*^2^ = 0.55 *P* < 0.01), but not T_c_ or T̄_sk_ (*R*^2^ = 0.07 and 0.37, respectively). Together, these observations appear to indicate that local temperature, independent of central temperature and hemodynamic reflexes, distinctly and noticeably influences limb blood flow during moderate passive heat stress.

Conflicting evidence over the past decades has rendered the role of temperature on functional hyperemia as highly contentious. Several previous studies have reported no change in exercise hyperemia during one-legged knee extensions (14, 51), two-legged cycling (51), or walking uphill to exhaustion in the heat (37), whereas our recent study showed significant increases in LBF during submaximal knee-extensor exercise across different levels of heat stress (up to 0.7 l/min) (42). These elevations are of a similar magnitude to the ∼0.5 l/min higher LBF observed in a previous study (14) when thigh muscle temperature was heated by ∼3°C before knee-extensor exercise, although the difference in flow was deemed not statistically significant in this earlier study. The small changes in temperature between heated and cooled conditions [∼0.3°C in (51)] and the use of an exercise protocol that engages a large muscle mass (37, 51) and thus markedly increases sympathetic vasoconstrictor drive (7, 34, 41, 46, 50) might explain the discrepant results. Here we hypothesized that if local temperature is important for functional hyperemia, an additive effect of both thermoregulatory and metabolic stimuli should result in further increases in blood flow during combined heat stress and exercise. In support of recent findings, the 0.6 l/min increase in heated leg blood flow seen with passive heat stress was maintained at near identical levels in all participants throughout incremental exercise to near maximal power output, with increases in leg vascular conductance once again being tightly coupled to femoral blood temperatures, as exercise intensity gradually increased (*R*^2^ = 0.95 and 0.85 for cooled and heated legs, respectively; *P* < 0.01 for both). Strikingly, LBF responses following isolated leg heating and cooling were virtually identical to those observed following systemic heat stress, despite differences in T_c_ and cardiac output of up to 1°C and 2 l/min before the commencement of exercise. Hence, these observations lend support to an independent and additive effect of thermoregulatory factors on LBF during heat stress and small muscle mass exercise.

### Mechanisms of Blood Flow Control in the Heat-Stressed Human Leg

Severe heat stress induces a hyperadrenergic state, characterized by augmented circulating catecholamines (29), enhanced muscle and skin sympathetic nerve activity (10, 38, 39), and hyperthermia and hyperkinetic hemodynamics at limb and systemic levels (47). A key integrative physiology question in this study was whether central neural and humoral reflexes drive local tissue blood flow responses to moderate heat stress or whether local thermosensitive mechanisms are more important. The virtual abolition of a hyperemic effect in the cooled leg in the face of significant increases in both systemic drive and opposing contralateral heated leg blood flow suggests a key role of local temperature-sensitive mechanisms, a notion supported by a similar magnitude increase in heated leg blood flow in the isolated limb thermal protocol.

The increased perfusion to different leg tissues with heat stress was associated with net vasodilatation, as indicated by the increases in conduit artery vascular conductance, irrespective of the perfusion pressure gradient response. Significant increases in skin blood flow during heat stress are well documented in the literature, and have been shown to be regulated by afferent-activated axon reflexes (54), NO (27, 28), and a sympathetically mediated component (9). Although observations from the current study confirmed these increases through significant increases in both laser Doppler-measured skin blood flow and superficial femoral artery blood flow (supplying extensive regions of superficial leg tissues), evidence for the presence of increased skeletal muscle perfusion suggests that these mechanisms do not entirely account for all of the increases in flow, however, as temperature stimuli also appears to affect skeletal muscle (22, 26). These findings confirm recent investigations into increased skeletal muscle perfusion during heat stress and, in this construct, suggest that temperature may be acting either directly or indirectly at the microcirculation level via potentiation of thermosensitive pathways. With respect to skeletal muscle, in vitro studies provide no evidence of direct vasoactive effects of temperature elevations on arterial and venous microvessel preparations from humans and canines (23, 55, 56). It therefore seems that temperature exerts its vascular effects through thermosensitive signal transduction pathways. Recent findings using isolated human skeletal muscle feed arteries have suggested a role for a heat-induced sympatholysis of α_1_ and α_2_ adrenoreceptors through the activation of TRPV channels (16). In addition to this potential mechanism, evidence from in vivo and in vitro studies suggest the involvement of ATP release from human erythrocytes in the regulation of tissue hyperemia in hyperthermic conditions (25, 42). The direct relationship between increases in erythrocyte ATP release and temperature (25) and the potent vasodilatory and sympatholytic properties of ATP in the human leg and arm circulations (17, 30, 46) make temperature-dependent erythrocyte ATP release an attractive mechanism. Another possibility based on the presently observed 64% increase in deep tissue V̇O_2_ in the heated leg is a metabolic contribution to hyperthermia-mediated hyperemia. Although the contributions of extracellular and intracellular themosensitive mechanisms remain to be fully established, the parallel decrease in deep tissue oxygen extraction and blood a-v glucose gradient, but increased limb tissue aerobic metabolism and glucose uptake (31), would point to potential intracellular signaling pathways playing a part in the metabolic stimulated vasodilatation.

### Experimental Considerations

The use of the Modelflow technique for the calculation of Q̇ has previously been reported to underestimate increases in systemic blood flow during heat stress conditions when compared with invasive thermodilution techniques (5). However, increases in Q̇ reported here when T_c_ increased 0.5 to 0.6°C are consistent with the rate of ∼3 l·min^−1^·°C^−1^ reported in previous literature (6, 15, 33, 36, 38, 40, 42, 43, 49) and are therefore considered to be representative of systemic blood flow. Great saphenous blood flow was not measured directly in this study, and therefore data involved in the calculations of leg V̇O_2_ were taken from a comparable protocol carried out in eight healthy males in a follow-up study. Methodological considerations prevented the counterbalancing of the heated and cooled legs during exercise in *Study 1*, with the cooled leg always being exercised first to maintain the highest possible local temperature difference between legs. Previous research has shown elevations in muscle blood flow and aerobic metabolism following repeated, maximal knee-extensor exercise bouts in humans, separated by a short recovery (2, 32). The current experimental design, however, used submaximal exercise to a level no greater than 80% of peak power output, with exercise bouts separated by 20 min and performed with different legs. Leg blood flows in *Study 2*, which used a counterbalanced experimental design, were identical to those observed in *Study 1*. This indicates that the additive increases in flow documented here during incremental exercise were associated with elevations in local tissue and blood temperatures, rather than the potentiating effects of previous exercise.

### Conclusion

This study provides compelling evidence that increases in limb tissue perfusion during passive heat stress and small-muscle mass exercise are tightly coupled to increases in local tissue and blood temperature, but clearly dissociated from the distinct systemic temperature and hemodynamic stimuli evoked by isolated leg and whole body heat stress. In addition, strong evidence is provided that skeletal muscle blood flow contributes to the observed hyperemic response, a response that may be in part due to increases in metabolic vasodilatation. These findings emphasize the importance of local temperature-sensitive mechanisms in the regulation of peripheral blood flow in hyperthermic humans and suggest a potential therapeutic use of local heating to improve oxygen and substrate delivery to specific tissues without the additional cardiac strain of whole-body hyperthermia. The additional observation that limb muscle glucose uptake and aerobic metabolism are potentiated in resting heat-stressed humans raises the possibility that local heating might also be beneficial in improving vascular and metabolic function in some patient populations with circulatory and metabolic diseases.

## GRANTS

The invasive study was partially funded by Gatorade Sports Science Institute, PepsiCo.

## DISCLAIMER

The views contained within this document are those of the authors and do not necessarily reflect those of PepsiCo.

## DISCLOSURES

No conflicts of interest, financial or otherwise, are declared by the author(s).

## AUTHOR CONTRIBUTIONS

Author contributions: S.T.C. and J.G.-A. conception and design of research; S.T.C., S.J.T., K.K., M.R., D.S.B., M.D.L., L.A., and J.G.-A. performed experiments; S.T.C. analyzed data; S.T.C., S.J.T., M.R., and J.G.-A. interpreted results of experiments; S.T.C. prepared figures; S.T.C. drafted manuscript; S.T.C., S.J.T., K.K., M.R., and J.G.-A. edited and revised manuscript; S.T.C., S.J.T., K.K., M.R., D.S.B., M.D.L., L.A., and J.G.-A. approved final version of manuscript.
